# Supplementary material for: Association between oral microbiome and seven types of cancers in East Asian population: a two-sample Mendelian randomization analysis
Source: Front Mol Biosci. 2023 Nov 21;10:1327893. doi: 10.3389/fmolb.2023.1327893 (PMC10702768; doi:10.3389/fmolb.2023.1327893)
Supplement: Supplementary file 1 [file DataSheet1.ZIP › Supplementary Materials/MR plots of saliva/saliva═╝/ovarian cancer/pheno.3258_to_ovarian cancer_leave_one_out.pdf]

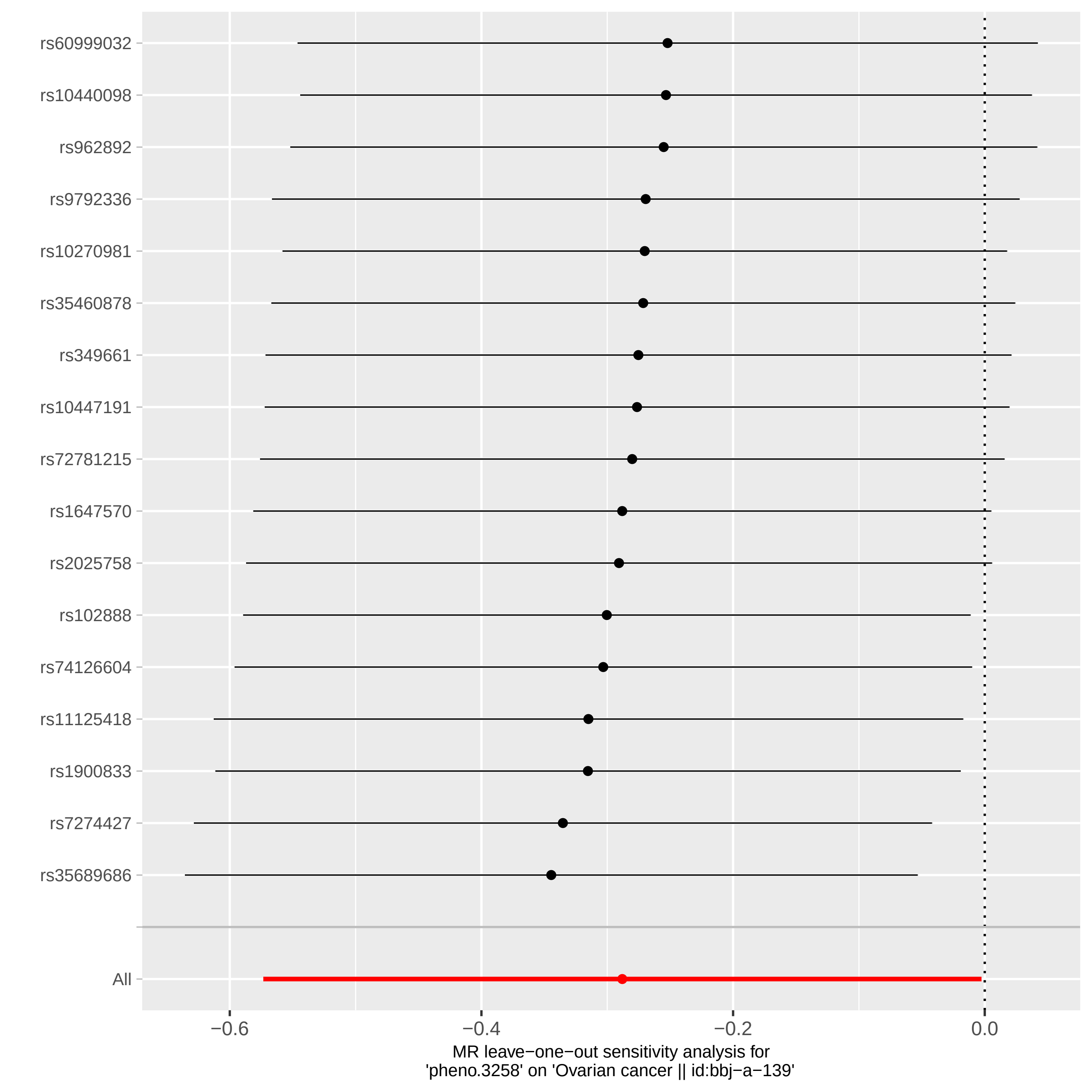

rs60999032
rs10440098
rs962892
rs9792336
rs10270981
rs35460878
rs349661
rs10447191
rs72781215
rs1647570
rs2025758
rs102888
rs74126604
rs11125418
rs1900833
rs7274427
rs35689686
All
−0.6
−0.4
−0.2
0.0
MR leave−one−out sensitivity analysis for
'pheno.3258' on 'Ovarian cancer || id:bbj−a−139'
